# Live-cell imaging probes to track chromatin modification dynamics

**DOI:** 10.1093/jmicro/dfab030

**Published:** 2021-08-13

**Authors:** Yuko Sato, Masaru Nakao, Hiroshi Kimura

**Affiliations:** Cell Biology Center, Institute of Innovative Research, 4259 Nagatsuta-cho, Midori-ku, Yokohama 226-8503, Japan; School of Life Science and Technology, Tokyo Institute of Technology, 4259 Nagatsuta-cho, Midori-ku, Yokohama 226-8501, Japan; School of Life Science and Technology, Tokyo Institute of Technology, 4259 Nagatsuta-cho, Midori-ku, Yokohama 226-8501, Japan; Cell Biology Center, Institute of Innovative Research, 4259 Nagatsuta-cho, Midori-ku, Yokohama 226-8503, Japan; School of Life Science and Technology, Tokyo Institute of Technology, 4259 Nagatsuta-cho, Midori-ku, Yokohama 226-8501, Japan

**Keywords:** live-cell imaging, epigenetics, histone modification, DNA methylation, fluorescence microscopy, intrabodies

## Abstract

The spatiotemporal organization of chromatin is regulated at different levels in the nucleus. Epigenetic modifications such as DNA methylation and histone modifications are involved in chromatin regulation and play fundamental roles in genome function. While the one-dimensional epigenomic landscape in many cell types has been revealed by chromatin immunoprecipitation and sequencing, the dynamic changes of chromatin modifications and their relevance to chromatin organization and genome function remain elusive. Live-cell probes to visualize chromatin and its modifications have become powerful tools to monitor dynamic chromatin regulation. Bulk chromatin can be visualized by both small fluorescent dyes and fluorescent proteins, and specific endogenous genomic loci have been detected by adapting genome-editing tools. To track chromatin modifications in living cells, various types of probes have been developed. Protein domains that bind weakly to specific modifications, such as chromodomains for histone methylation, can be repeated to create a tighter binding probe that can then be tagged with a fluorescent protein. It has also been demonstrated that antigen-binding fragments and single-chain variable fragments from modification-specific antibodies can serve as binding probes without disturbing cell division, development and differentiation. These modification-binding modules are used in modification sensors based on fluorescence/Förster resonance energy transfer to measure the intramolecular conformational changes triggered by modifications. Other probes can be created using a bivalent binding system, such as fluorescence complementation or luciferase chemiluminescence. Live-cell chromatin modification imaging using these probes will address dynamic chromatin regulation and will be useful for assaying and screening effective epigenome drugs in cells and organisms.

## Introduction

**Fig. 1.**
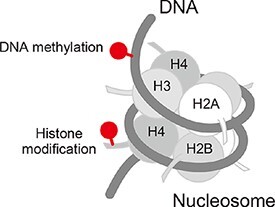
Nucleosome and the modifications. DNA wraps around core histone proteins (H2A, H2B, H3 and H4) to form a nucleosome. Epigenetic modifications including DNA methylation and histone posttranslational modifications (indicated by red lollipops) play fundamental roles in chromatin organization.

In eukaryotic nuclei, DNA wraps around histone proteins to form a nucleosome, a basic structural unit of chromatin (Fig. 1). Each nucleosome core contains a histone octamer formed by two copies of four core histone proteins (H2A, H2B, H3 and H4) and stably binds to DNA. The nucleosome structure as such can be an obstacle to inhibit gene regulatory factors from accessing DNA. However, the nucleosome structure is dynamically altered by its effector molecules, which leads to conformational changes in chromatin so that the nucleosome array can function as an allosteric scaffold for genome function.

A mechanism that regulates chromatin organization and dynamics involves the post-translational modification of histones, such as phosphorylation, acetylation, methylation and ubiquitination. The best-characterized histone modifications are acetylation and methylation of lysine residues on the N-terminal tails of histones H3 and H4. Lysine acetylation is correlated with active transcription, whereas lysine methylation is associated with either transcription activation or repression, depending on the site and the degree of methylation from one to three [1,2]. The positive charge of the lysine residue is neutralized by acetylation, which can loosen the DNA–histone contact, but the charge is not affected by methylation. The modifications are recognized by effector molecules, which play key roles in genome function, such as transcription, DNA replication and DNA damage repair.

There has been a great effort to elucidate how histone modifications regulate gene expression and how specific modifications are regulated during development and differentiation. As many modifying enzymes can be responsible for modifications at different sites on histones and non-histone proteins, and a modification can be added by multiple enzymes, it is not straightforward to interpret a result by loss-of-function experiments of an enzyme as a function of the specific modification. Therefore, it has been challenging to reveal the biological function of a specific modification.

In higher eukaryotes, DNA modifications play an important role in gene regulation [3]. The most common DNA modification in the mammalian genome is cytosine methylation in a symmetrical CpG (5′-C-phosphate-G-3′) context. DNA CpG methylation is generally associated with transcriptional repression and is involved in numerous biological processes, including transposon repression, genomic imprinting and X chromosome inactivation. Recent studies have shown that the methylation undergoes more dynamic changes than previously thought, especially during early development [4].

**Fig. 2.**
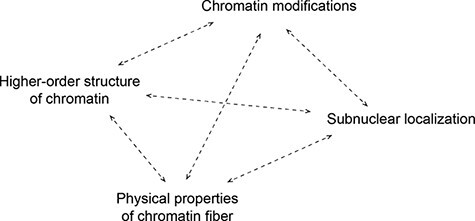
Interplay between epigenetic factors regulating chromatin organization. Chromatin modification is involved in the spatiotemporal organization of the genome at different levels and regulates genome-related events.

Chromatin modifications are involved in the spatiotemporal organization of the genome at different levels, including the physical and biochemical properties that affect the flexibility and compaction of nucleosome arrays, higher-order chromatin structures and subnuclear localization (Fig. 2). The organizations at different levels are related to each other and regulate gene expression and genome integrity. Insights into the organization and dynamics of chromatin have been obtained by epigenome analysis and microscopy. The recent advance of new optics like superresolution systems and computer-assisted image analysis has made it possible to reveal fine and dynamic structures in the fluorescence microscopy approach. In addition, the development of live-cell probes has also been essential to track chromatin and its modifications in living cells [5,6]. In this review, we introduce various probes for visualizing chromatin and its modifications under a fluorescence microscope and discuss the principles, advantages and limitations of the probes in different categories. We also mention the application of the various probes for biology and medicine.

## Visualizing chromatin in living cells

### Bulk labeling of chromatin

Chromatin can be visualized in bulk by using DNA-staining dyes and by expressing histones tagged with a fluorescent protein (FP). By bulk labeling, the distribution of chromatin can be observed, and densely stained regions essentially represent condensed heterochromatin. A popular DNA staining dye, Hoechst 33342, binds preferentially to AT-rich sequences, and so, it highlights AT-rich heterochromatin better than histone–FP. Hoechst 33342 is cell-permeable and convenient to use in many cell types without genetic manipulation [7]. A drawback of using Hoechst dye is its short excitation wavelength (ultraviolet to 405 nm) so that phototoxicity from repeated excitation could cause apoptosis [8]. Hoechst-tagged long-wavelength excitable dyes have been developed [9,10], although the use of these dyes still needs caution to avoid any toxicity [11].

To label nucleosomes, histone H2B–FP has been widely used to detect nuclei and chromosomes in living cells [12]. Once stable lines are generated, chromatin can be tracked over cell generations and during development and differentiation [13,14]. Histone H2B–FP has also been used for analyzing nucleosome stability and chromatin movements [e.g. ref 15–17]. Single-molecule analysis based on stochastic labeling of nucleosomes by illuminating small amounts of fluorophores on histones has been a strategy to analyze local nucleosome fluctuations in relation to biological processes [16]. It has been shown that chromatin is globally stabilized by loose connections through active transcription machinery [17].

Besides the incorporation of histone-FP, nucleosomes can also be labeled with nucleosome-binding molecules. A number of cellular and virus proteins are known to bind to the acidic patch of H2A–H2B in a nucleosome [18]. N-terminal region of latency-associated nuclear antigen (LANA) from Kaposi’s sarcoma-associated herpesvirus is one of those proteins [19]. Although a short peptide is subjected for degradation, conjugating with polyethylene glycol (PEG) stabilizes LANA in living cells. Thus, fluorescently labeled PEG–LANA can be used for bulk labeling, although its injection into cells is needed [20]. A genetically encoded FP-tagged nanobody that binds to the acidic patch, or chromatibody, has also been developed as a replacement for histone–FPs [21].

### Labeling specific genomic loci

To visualize specific genomic loci in living cells, several systems have been developed. As a fluorescent focus requires detectable brightness over background signals, the fluorescent reporter needs to be concentrated at the locus. Fluorescent reporter–operator systems are based on the insertion of an array of bacterial operator sequences (e.g. LacO, TetO and LambdaO) to which a corresponding FP-tagged repressor protein (e.g. LacI, TetR and LambdaR) binds [22–24]. The insertion of artificial tandem operator repeats, however, could induce the accumulation of DNA methylation [25], and inserting multicopy binding sequences to a specific locus is often challenging. To avoid inserting repeat sequences and blocking transcription and replication, the system using ParB and its binding sequence, called ANCHOR, has been developed [26,27]. As the ANCHOR system employs the oligomerization nature of the binding protein, the insertion sequences can be small, less than 1 kb, non-repetitive and non-disruptive of chromatin structure.

Instead of inserting exogenous DNA sequences and expressing corresponding binding proteins, endogenous genomic loci can now be visualized using artificial DNA binding proteins derived from genome editing systems, including zinc finger (ZF) proteins [28], transcription activator-like effectors (TALEs) [29,30] and clustered regularly interspaced short palindromic repeat and nuclease-dead Cas9 (CRISPR/dCas9) [31,32]. These probes allow for the visualization of not only repetitive sequence targets like pericentromeric and telomeric regions but also unique loci [31,33]. Chemically synthesized molecules (e.g. peptide nucleic acids and pyrrole-imidazole polyamides) that specifically bind to pericentromeric and telomeric repeats have also been developed [34–37]. These probes are flexible, can be conjugated with various chemical dyes and do not require genome modifications, which might make them suitable for high-throughput analysis and diagnostics.

### Probes to visualize chromatin modifications

**Fig. 3.**
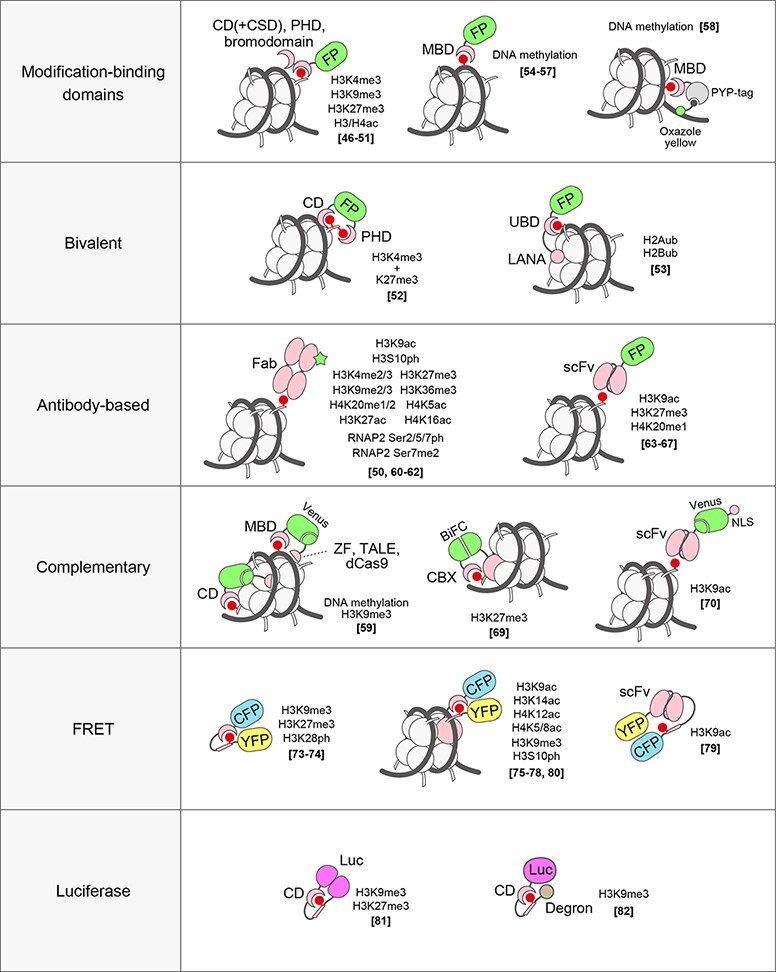
Live-cell chromatin modification probes and sensors. Live-cell probes and sensors are categorized by the recognition and reporter systems. Target modifications and references are shown.

To detect the distribution of chromatin modifications and their dynamics in living cells, several types of probes have been developed (Fig. 3).

#### Probes using modification-binding domains of reader proteins

Histone modifications are recognized by their reader proteins through specific binding domains, such as chromodomains (CDs) and plant homeodomains (PHDs) for methylated lysine residues and bromodomains for acetylated lysine residues [38–44]. Therefore, such binding domains in principle can be used as live-cell probes by fusing them with FP to detect the modifications. However, the binding affinities of these binding domains to their target modifications are rather low (at the micromolar order for the dissociation constant), and so engineering bivalent binding proteins is often necessary. For example, heterochromatin protein 1 (HP1) forms a dimer through its chromoshadow domains (CSDs) and directly binds to H3K9me3 through its CD to function in gene silencing [38,45]. The full-length HP1 and fragments containing a CD and a CSD have been used to visualize heterochromatin domains in living nuclei [46–49]. To visualize the dynamics of bromodomain proteins in living zebrafish embryos, GFP (Green Fluorescent Protein)-tagged bromodomains from human BRD4 were used [50]. Synthetic probes consisting of tandem modification-binding domains have enabled the identification of proteins associated with specific modifications, such as H3K27me3 (with CBX7 and Drosophila Polycomb CD), H3K9me3 (with CBX1 CD) and H3K4me3 (with TAF7 PHD) [51]. In the method called ChromID, biotin ligase-fused synthetic modification-binding domains were expressed in living cells, and proteins around the target modifications were biotinylated for affinity purification followed by mass spectrometry. By fusing these synthetic domains with FP, the localization of the target modifications was visualized in living cells [51], providing a possibility for tracking the modifications.

Connecting two different binding domains with weak affinities has also been used to develop unique probes. By fusing Polycomb CD that binds to H3K27me3 and TAF3 PHD that binds to H3K4me3 at the N-terminus and C-terminus of an FP, bivalent nucleosomes that have both modifications were visualized [52]. A probe that detects ubiquitinated H2A and H2B has been developed by linking a ubiquitin-binding domain to LANA [53].

For visualizing DNA methylation, an FP-tagged methyl-CpG-binding domain (MBD) of the MBD1 protein is widely used [54,55]. Transgenic animals expressing the DNA methylation probe were also generated to study DNA methylation dynamics during early development [56,57]. A hybrid probe using a DNA-enhanced fluorescence dye and MBD has also been developed for background-free detection of DNA methylation in living cells [58]. This probe contains MBD and a protein tag (PYP-tag), which is labeled with an oxazole yellow-conjugated ligand. Oxazole yellow is less fluorescent when not bound to DNA and its DNA binding affinity is low, but when the probe binds to methyl-CpG through its MBD, the oxazole yellow moiety binds to nearby DNA and becomes fluorescent. Therefore, this probe can highlight methyl-CpG without a diffuse background due to unbound probe molecules [58].

Modifications at specific genome loci have also been detected using a combination of a sequence-specific DNA binding module (ZF, TALE or CRISPR/dCas9) and a modification binder (MBD or HP1 CD) tagged with the N-terminus and C-terminus of Venus, respectively [59]. Only when the target chromatin is modified does a reconstituted Venus form and fluoresce.

#### Antibody-derived probes

**Fig. 4.**
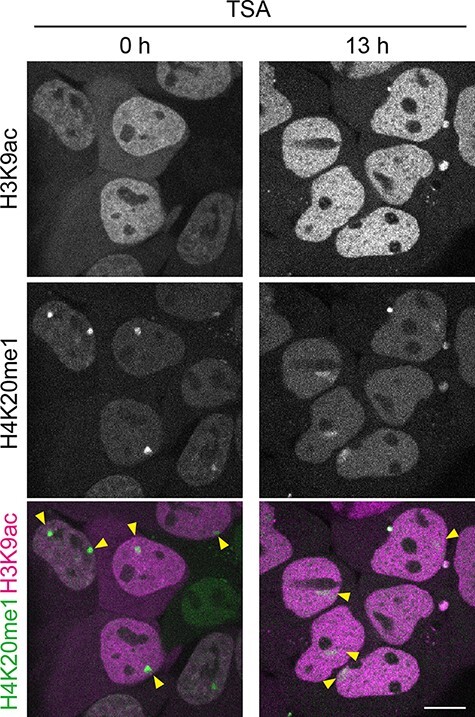
Live-cell imaging of H3K9ac and H4K20me1 using Mintbodies. H3K9ac-mintbody (EGFP version; magenta) and H4K20me1-mintbody (mCherry version; green) were expressed in MC12 mouse carcinoma cells that harbor one or two inactive X chromosomes (Xi, indicated by yellow arrowheads). The distribution of Mintbodies is representative of the concentration of the target modifications, such as H3K9ac-mitbody on euchromatin and H4K20me1-mintbody on Xi. In addition to the chromatin-bound molecules, chromatin-free Mintbody molecules are present in both the nucleus and cytoplasm. Upon the addition of an HDAC inhibitor, trichostatin A (TSA), H3K9ac-mintbody is more accumulated in the nuclei with its decrease in the cytoplasm (13 h) because the chromatin-free molecules that diffuse into the cytoplasm are decreased by the increase of the target acetylation on chromatin. In contrast, the enrichment of H4K20me1 on Xi was decreased and the Xi foci appeared blurred, possibly due to Xi decondensation and/or decreased level of methylation, induced by increased levels of acetylation. Scale bar, 10 μm.

In fixed cells, histone modifications are usually detected with specific antibodies. In living cells, using the antibody to visualize modifications is challenging because (1) it is not straightforward to deliver IgG molecules into cells, (2) the loaded immunoglobulin G (IgG) is too big to pass through nuclear pores and (3) the binding affinity (at the picomolar to nanomolar order for the dissociation constant) may be too strong and block the access of endogenous binding proteins to the target modification. In contrast to the whole IgG, fluorescently labeled antigen-binding fragments (Fabs) have been shown to be suitable for detecting endogenous histone modifications in living cells and embryos [50,60–62]. Unlike IgGs, Fabs are small enough to pass through the nuclear pore, and their affinity is on the sub-micromolar to micromolar order (for the dissociation constant), i.e. 10- to 1000-fold lower than full-length IgG. The residence time of Fab on target modifications in living cells is usually a few seconds so that endogenous proteins can bind to the modification when needed. For this reason, Fab-loaded cells progress through the cell cycle and Fab-loaded embryos develop normally. Genetically encoded probes derived from antibody coding sequences have also been developed. The antibody single-chain variable fragment (scFv) tagged with an FP or modification-specific intracellular antibody (Mintbody) can be used for long-term time-lapse and *in vivo* imaging by establishing stable cell lines and transgenic animals (Fig. 4) [63,64]. As the binding affinity and residence time of Mintbodies are similar to those of Fabs, endogenous proteins can still bind to the target modifications. The low toxicity of Mintbody has been demonstrated by the viability and fertility of transgenic animals and plants that express Mintbody being viable and fertile [63,65–67]. Although genetically encoded Mintbodies are more convenient to use than Fabs, so far only a limited number of probes (for H3K9ac, H3K27me3 and H4K20me1) are available. This is because the successful cytoplasmic expression of antibody scFv depends on its proper folding and stability [63–65,68].

#### FRET-based probes

In living cells, the probes described above are present at least in two fractions, bound and unbound to the target modifications. The location of modifications is thus evaluated by the enrichment of probes over the diffuse background fluorescence. If the affinity of the probe is relatively low or the target modification is less abundant, the signal at the modification sites can be ambiguous because the unbound molecules that diffuse freely in the nucleus are present at high levels. Increasing the probe affinity can reduce the background from free molecules, but this may block the binding of endogenous proteins. It is therefore ideal if the probe becomes fluorescent only when binding to the target. A FP complementation strategy can be used for this purpose to reconstitute FPs at the site of modifications [59,69,70], but the reconstituted FPs are stable once complemented and so this irreversibility makes the kinetic measurement difficult. A reversible fluorescence reporter, such as a Flashbody, appears to be more suitable [71], although the construction of such a reporter may not be straightforward. Ratiometric measurements based on fluorescence/Förster resonance energy transfer (FRET) can partly address the background issue. Unlike the binding probes that highlight the endogenous modification, the change of modification on FRET sensors can be used to monitor the response to the balance between modifying and de-modifying enzymes.

A FRET sensor usually consists of a modification site and a modification-binding domain in between a donor and an acceptor FP [72]. The very first epigenetic FRET sensors were designed for histone methylation with HP1 or Polycomb CD and phosphorylation with 14-3-3τ in combination with histone and an H3 N-terminal peptide [73,74]. As these probes do not have histone fold regions, they are unlikely to be incorporated into nucleosomes. Chromatin-bound FRET sensors harboring a full-length histone protein have therefore been developed to monitor acetylation, methylation and phosphorylation in a more natural setting. A series of Histac FRET sensors detect changes in the levels of H3K9ac, H3K14ac, H4K12ac and H4K5/8ac [75–78]. These sensors, consisting of an H3 or H4 histone, a bromodomain and a pair of FPs, have been shown to be useful for screening and evaluating chemical drugs that target histone acetyltransferases, histone deacetylases (HDACs) and acetyl-reader bromodomain and extraterminal domain (BET) family proteins, which are associated with the onset of aggressive cancers. A FRET sensor using H3K9ac-specific scFv as the binding module to acetylation has also been shown to sensitively detect the effect of HDAC inhibitors [79]. The dynamic changes of histone H3K9 trimethylation and H3S10 phosphorylation were monitored by FRET sensors using HP1 CD and yeast Rad53 FHA2 phosphothreonin-binding domain, respectively, as the modification binding modules [80]. By using the CFP-YFP and LLSmOrange-FusionRed FRET pairs, both H3K9me3 and H3S10ph levels were detected simultaneously.

#### Luciferase-based probes for *in vivo* imaging

For *in vivo* imaging, a split-Renilla luciferase (Rluc) complementation system has been applied for histone methylation [81]. In this system, the N-terminal fragment of Rluc fused with Suv39h1 CD and the C-terminal fragment of Rluc fused with H3 N-terminal amino acids (1–13) were co-expressed in cells. When the ninth lysine residue is methylated, Rluc activity is expected to be reconstituted because the N- and C-fragments can get close due to the binding of Suv39h1 CD. By injecting cells that express the reporters into mice, the luciferase activity can be monitored *in vivo*. A degron blockade methylation sensor was also developed for luciferase-mediated methylation sensing [82]. In this case, a full-length firefly luciferase is fused with the H3 N-terminal amino acids, a linker, Suv39h1 CD and a degron protease recognition sequence. When the ninth lysine residue is methylated, Suv39h1 CD binds to the methylation and induces a conformational change to block degron recognition. Without methylation, the sensor is subjected to degradation. The luciferase activity can also be monitored in mouse models.

## Applications and conclusions

By using these modification-specific live-cell probes, the distribution and changes of modifications on DNA, histones and other chromatin proteins can be tracked in living cells. One of the applications of live-cell chromatin modification imaging is to reveal the role and function of modifications. There has been a long-standing question on chromatin modifications—are they regulators or just passive by-products of genome function? Especially in the field of transcriptional regulation, it has been elusive whether active marks of chromatin (e.g. acetylated histones) can activate transcription or are added as a consequence of transcription [83,84]. To distinguish cause from effect, spatiotemporal information from live-cell imaging can provide critical evidence. Single-cell live-imaging using fluorescent Fabs specific to histone H3K27 acetylation with the active transcription mark (phosphorylated RNA polymerase II) revealed that this histone modification precedes transcription activation [50,62]. In another example, by using cells expressing two Mintbodies specific to H3K27me3 and H4K20me1, a concurrent accumulation of the two modifications on inactivated X chromosomes was observed [63]. Combined with epigenome analysis, a shared recruitment mechanism and distinct function in the two modifications were suggested.

Another major application of live-cell modification imaging is to monitor the effect of drugs. As epigenome drugs are expected to be a new class of agents against cancers and other diseases [85], a convenient and reliable assay to evaluate the action of drugs at cellular and animal levels is demanding. The FRET-based ratiometric assay may be particularly suitable for this purpose, and luciferase-based probes have opened an avenue toward *in vivo* analysis [86].

We anticipate that the use of these probes described above will be broadly applicable and shed light on the intricate connections between chromatin modifications and genome functions, as well as for screening effective epigenome drugs.
